# 2638. Patterning of high-risk comorbidity associated with vaccination but not influenza status among hospitalized patients in an influenza test-negative design cohort

**DOI:** 10.1093/ofid/ofad500.2250

**Published:** 2023-11-27

**Authors:** Aleda M Leis, Arnold Monto, Emily T Martin

**Affiliations:** University of Michigan School of Public Health, Ann Arbor, Michigan; University of Michigan, Ann Arbor, MI; University of Michigan, Ann Arbor, MI

## Abstract

**Background:**

The test-negative design is common in studies of influenza vaccine effectiveness. However, estimates are biased when there is heterogeneity in healthcare seeking behavior by vaccination or case status, both influenced by presence of high risk comorbidities. This study sought to examine potential differences in case rate and vaccination status by patterning of these comorbidities among hospitalized patients enrolled during the 2018/19 influenza season.

**Methods:**

Participants were hospitalized with acute respiratory illness during the 2018/2019 influenza season in three major hospital systems in southeast Michigan within the US Hospitalized Adult Influenza Vaccine Effectiveness Network (HAIVEN). Influenza was confirmed by molecular testing of respiratory specimens. Latent class models with 4 classes were constructed from 17 ICD-10 diagnostic code defined high risk conditions to assess clustering among individuals; this analysis assumes latent similarities exist among those within a class defined using known conditions. Individuals were assigned the class with the highest membership posterior predicted probability. Chi-square tests were used to compare classes.

**Results:**

Characteristics of latent classes are in **Figure 1** (n=840). There were statistically significant differences between classes for all conditions; Class 2 had highest prevalence of comorbidity with high frequencies of cardiometabolic and lung disease, and Class 3 had lowest prevalence of comorbidity with only modest frequency of lung disease. Influenza cases represented 18.4%, 13.6%, 20.5%, and 18.3% of those in each class, respectively (p > 0.05 for all pairwise comparisons). Vaccination rates were 77.6%, 77.3%, 55.8%, and 67.6%, respectively, with statistically significant differences between all classes except Classes 1 and 2 (p < 0.05 for all other comparisons).

**Figure 1.**
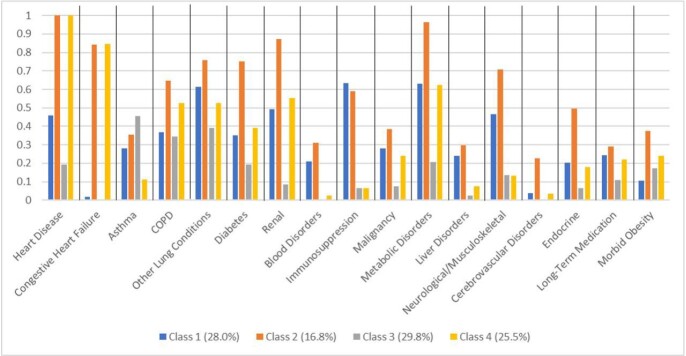
Conditions included in the latent class construction and subsequent frequencies in the assigned class. Total n=840.

**Conclusion:**

Latent class analysis identified significant variation in influenza vaccination rates by patterning of high risk comorbidity among adults hospitalized with acute respiratory illness. Care should be taken to examine such differences within test-negative studies of vaccine effectiveness in hospitalized populations, with particular concern during circulation of multiple vaccine-preventable respiratory viruses.

**Disclosures:**

**Arnold Monto, MD**, Roche: Advisor/Consultant|Roche: Honoraria **Emily T. Martin, PhD, MPH**, Merck: Grant/Research Support

